# A Potential Application of Dynamic Contrast-Enhanced Magnetic Resonance Imaging Combined with Photodynamic Diagnosis for the Detection of Bladder Carcinoma in Situ: Toward the Future ‘MRI-PDD Fusion TURBT’

**DOI:** 10.3390/diagnostics9030112

**Published:** 2019-09-04

**Authors:** Makito Miyake, Fumisato Maesaka, Nagaaki Marugami, Tatsuki Miyamoto, Yasushi Nakai, Sayuri Ohnishi, Daisuke Gotoh, Takuya Owari, Shunta Hori, Yosuke Morizawa, Yoshitaka Itami, Takeshi Inoue, Satoshi Anai, Kazumasa Torimoto, Tomomi Fujii, Keiji Shimada, Nobumichi Tanaka, Kiyohide Fujimoto

**Affiliations:** 1 Department of Urology, Nara Medical University, 840 Shijo-cho, Kashihara, Nara 634-8522, Japan; 2 Department of Radiology, Nara Medical University, 840 Shijo-cho, Kashihara, Nara 634-8522, Japan; 3 Department of Diagnostic Pathology, Nara Medical University, 840 Shijo-cho, Kashihara, Nara 634-8522, Japan; 4 Department of Pathology, Nara City Hospital, 1-50-1 Higashi kidera-cho, Nara, Nara 630-8305, Japan

**Keywords:** bladder cancer, carcinoma in situ, contrast-enhanced magnetic resonance imaging, 5-aminolevulinic acid, photodynamic diagnosis

## Abstract

The detection of carcinoma in situ (CIS) is essential for the management of high-risk non-muscle invasive bladder cancers. Here, we focused on dynamic contrast-enhanced magnetic resonance imaging (DCE-MRI) combined with photodynamic diagnosis (PDD) for the detection of CIS. A total of 45 patients undergoing pre-surgical DCE-MRI and PDD-assisted endoscopic surgery accompanied by biopsies of the eight segmentations were analyzed. Immunohistochemical analysis of the biopsies revealed hypervascularity of CIS lesions, a cause of strong submucosal contrast-enhancement. It was found that 56 (16.2%) of 344 biopsies had pathologically proven CIS. In the DCE-MRI, the overall sensitivity and specificity for detecting CIS were 48.2% and 81.9%, respectively. We set out two different combinations of PDD and DCE-MRI for detecting CIS. Combination 1 was positive when either the PDD or DCE-MRI were test-positive. Combination 2 was positive only when both PDD and DCE-MRI were test-positive. The overall sensitivity of combinations 1 and 2 were 75.0% and 37.5%, respectively (McNemar test, vs PDD alone; *p* = 0.041 and *p* < 0.001, respectively). However, the specificity was 74.0% and 91.7%, respectively (vs PDD alone; both *p* < 0.001). Our future goal is to establish ‘MRI-PDD fusion transurethral resction of the bladder tumor (TURBT), which could be an effective therapeutic and diagnostic approach in the clinical management of high-risk disease.

## 1. Introduction

Bladder cancer (BCa) is one of the most common malignancies in both male and female patients, with approximately 430,000 new cases diagnosed annually worldwide [1]. Approximately 75% of the patients treated by transurethral resection of the bladder tumor (TURBT) present with non-muscle invasive bladder cancer (NMIBC) [2,3]. Despite recent advancements in multidisciplinary approaches, the detection and clinical management of high-risk NMIBC, especially of carcinoma in situ (CIS), is still challenging and problematic [4,5].

The presence of concurrent CIS is considered an important prognostic factor in the guidelines [6,7,8]. Because CIS is not curable by an endoscopic surgery alone, diagnosis of CIS must be followed by further treatment, either intravesical administration of Bacillus Calmette-Guérin (BCG) or radical cystectomy. Without any treatment, about half of the patients with CIS will progress to muscle-invasive disease [9]. Thus, the sensitive and early detection of CIS is essential for adequate management of the high-risk subset. Endoscopic detection technologies have been developed to visualize small tumors and flat lesions including CIS. Photodynamic diagnosis (PDD) with fluorescent cystoscopy using an excitation wavelength of 405 nm is performed after administration of 5-aminolevulinic acid (ALA) or hexaminolaevulinic acid. The fluorescence-guided biopsy and resection show better sensitivity compared to the conventional white-light procedure for the detection of tumors, particularly for CIS [3,10,11,12]. The major disadvantage of ALA-PDD is relatively low specificity and positive predictive value (PPV). A long-term investigation by Hungerhuber et al. reported false-positive findings in 1769 (38.2%) out of 4630 biopsy specimens obtained by PDD-assisted TURBT (PDD-TURBT) [13]. The false-positive result is associated largely with the urinary mucosal inflammation and oblique illumination of the bladder wall with blue light [14,15,16,17,18]. Overestimation can lead to unnecessary resection, an increase in the rate of adverse events, and delay in the initiation of adjuvant intravesical treatment.

Imaging modalities such as transurethral endoscopic ultrasonography, computed tomography (CT), and magnetic resonance imaging (MRI) plays an important diagnostic role in the staging of BCa. Dynamic contrast-enhanced MRI (DCE-MRI) provides superior accuracy over CT for local staging of BCa [19,20]. To date, most studies have focused on the diagnosis of the tumor invasion depth (≤T1 vs ≥T2, ≤T2 vs T3, and <T4b vs T4b) [21]. To our knowledge, very few studies have investigated the potential of DCE-MRI to diagnose bladder CIS. To avoid overestimation and underestimation of bladder flat lesions, the present study reports a potential application of DCE-MRI combined with ALA-PDD for the detection of bladder CIS.

## 2. Materials and Methods

### 2.1. Data Collection of the Patients

The Nara Medical University Research Ethics Committee approved this study (the project identification code: 1966, the date of approval: 10 July 2018), and all the participants provided informed consent. The study was conducted in compliance with the study’s protocol and following the provisions of the Declaration of Helsinki (2013).

**Figure 1:**
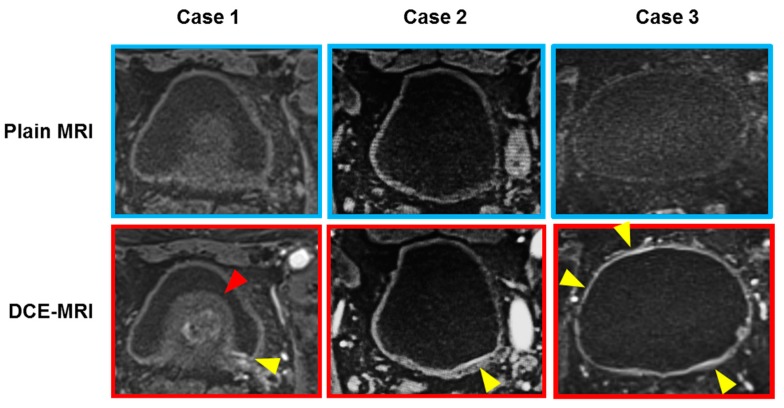
Three representative cases with flat lesions suspected bladder carcinoma in situ (CIS). Heterogeneous and blurry contrast-enhancement of the bladder mucosa and the submucosa (lamina propria) at the arterial phase of DCE-MRI is considered as a case of bladder CIS (yellow arrowheads). Case 1: A 71-year-old man with T4 high-grade urothelial carcinoma with CIS. DCE-MRI shows submucosal enhancement around the left ureteral orifice, which was pathologically proven CIS. The suspected CIS lesion (yellow arrowhead) was difficult to look at with the cystoscopy because of the enlarged benign prostate (red arrowhead). Case 2: An 88-year-old man with an isolated CIS on the posterior wall. Case 3: A 70-year-old woman with broad-range CIS in the whole bladder wall.

A total of 142 Japanese patients with suspected NMIBC underwent ALA-PDD assisted TURBT between June 2006 and July 2018. A total of 65 patients (46%) who did not undergo DCE-MRI before the surgery and 32 patients (22%) who had insufficient clinicopathologic data were excluded. The remaining 45 (32%) patients were included in this study (Figure 1). The clinical information was reviewed using the medical charts. The hematoxylin and eosin-stained specimens obtained by TURBT were reassessed independently by two experienced uropathologists (T.F. and K.S.) who were blinded to the original report for T category (2010 American Joint Committee on Cancer TNM Staging system), tumor grade (2004 WHO classification), and presence of CIS.

### 2.2. Procedure of ALA-PDD Assisted TURBT

ALA was dissolved in water immediately before the oral administration (20 mg/kg body weight) and was administered 3 h (allowable range: 2–4 h) before the initiation of TURBT. Cystoscopy examination and tissue collection were carried out as described previously [3,10]. The bladder was segmented into eight regions as shown in Figure 1. The bladder tumors and the intact regions were initially examined with a conventional white-light (WL) source, followed by careful inspection under a fluorescence (FL) source. The WL- and FL-based findings of all the eight segmented regions were recorded. A test-positive for the WL source was defined based on the published general rule [22]. A test-positive for the FL source was defined when the tissue emitted an abnormal red fluorescence. TUR or targeted cold-cup biopsy was performed to remove tissues that were identified as malignant lesion/test-positive using the WL and/or FL source. The cold-cup biopsies of the flat mucosa of all the eight segmented regions were performed and subjected to the pathological examination.

### 2.3. Immunohistochemical Staining

Immunohistochemical (IHC) staining of the paraffin-embedded, formalin-fixed tissue blocks were performed using the Histofine SAB-PO kit (Nichirei Co., Tokyo, Japan) as previously described [23]. The primary antibodies were rabbit polyclonal anti-PECAM-1 (CD31) (dilution 1/5000; Ref. sc-1506, Santa Cruz Biotechnology), rabbit polyclonal anti-VEGF (dilution 1/500; Ref. sc-152, Santa Cruz Biotechnology), and mouse monoclonal anti-Ki-67 (ready-to-use; clone MIB-1, Dako Japan). Bright-field images were captured using the EVOS^®^ FL Auto microscope (Life Technologies, Carlsbad, CA, USA). PECAM-1-stained vessels in the cancerous stromal area were counted in at least three independent fields (200× magnification). To quantify the expression level of VEGF and Ki-67, immunoreactive tumor cells were counted in at least three independent fields, and the percentage of positive cells was calculated by dividing that number by the total number of the counted cancer cells (1–100%). The evaluation was carried out by two investigators (T.O. and S.H) blindly, without knowledge of the patients’ clinicopathologic features.

### 2.4. DCE-MRI Scan Protocol

This retrospective study included patients who received the bladder MRI scan between 2006 and 2018. The scan protocol, scanners, and gadolinium contrasts changed with the time and, therefore, varied among the patients. Most of the patients were scanned according to the protocol as follow.

The patients were not allowed to urinate for ≥1 h before DCE-MRI to moderately distend the bladder. MR scans were obtained with a 1.5 T Magnetom Avanto scanner (Siemens Medical Solutions, Erlangen, Germany) or with a 3.0 T Magnetom Skyra scanner. Conventional T1-weighted spin-echo images and T2-weighted turbo spin-echo images were obtained. Subsequently, DCE images were acquired during the arterial phase (30 s) in the axial plane, followed immediately by the venous phase. A bolus injection of 0.1 mmol/kg gadopentetate dimeglumine (Gd-DTPA; Magnevist, Bayer) or Gadobutrol (Gd-BTDO3A; Gadvist, Bayer) was administered using a power injector at a rate of 3 mL/s followed by a 20 mL saline flush through the peripheral vein. The acquisition time was 30, 60, 90, 120, 150, and 180 s after the injection of the contrast agent. Sagittal and coronal images were added depending on the tumor location and the presence of tumor stalk.

### 2.5. Image Interpretation for Bladder Lesions

MR images were reviewed and interpreted by an MR radiologist (N.M., more than 20 years of experience) with special expertise in urogenital imaging. The investigator was blinded to the final pathological staging and presence of CIS obtained by TURBT or radical cystectomy. The reviewer assigned a radiologic stage according to the criteria previously described in the literature [24,25]. Also, we focused on the radiographic evaluation of the flat lesions suspected to be the bladder CIS. A Japanese report [26] demonstrated that the presence of bladder CIS is suspected when heterogeneous and blurry contrast-enhancement of the bladder mucosa and the submucosa (lamina propria) at the arterial phase of DCE-MRI is observed. DCE-MRI imaging of the representative cases is shown in Figure 1. This observation and phenomenon can be considered different from the submucosal linear enhancement, which was homogenous, clear, and sharp contrast-enhancement of bladder submucosa localized at the base of the tumor [27]. Based on multi-planar reconstruction, including axial, sagittal, and coronal DCE-MRI images, the location of CIS-suspected flat lesion was recorded for each segmentation area.

### 2.6. Statistical Analysis

The Wilcoxon signed-rank test was used to analyze the difference in the number of vessels in the stromal area, VEGF-positive cancer cells, and proliferative cancer cells among the matching surgical specimens. Sensitivity, specificity, positive predictive value (PPV), and negative predictive value (NPV) for detection of CIS in each test were determined by pathologic confirmation of CIS in all specimens according to standard methods. The sensitivity and specificity among the tests were compared using the McNemar test. McNemar’s test was used to assess the significance of differences in paired data. IBM SPSS Version 21 (SPSS Inc., Chicago, IL, USA) and PRISM software version 7.00 (GraphPad Software Inc., La Jolla, CA, USA) were used for statistical analyses and plotting the data, respectively. Statistical significance in this study was set at *p* < 0.05 and all reported *p* values were two-sided.

## 3. Results

### 3.1. Patient Characteristics and Tissue Specimens Obtained from TURBT

**Figure 2:**
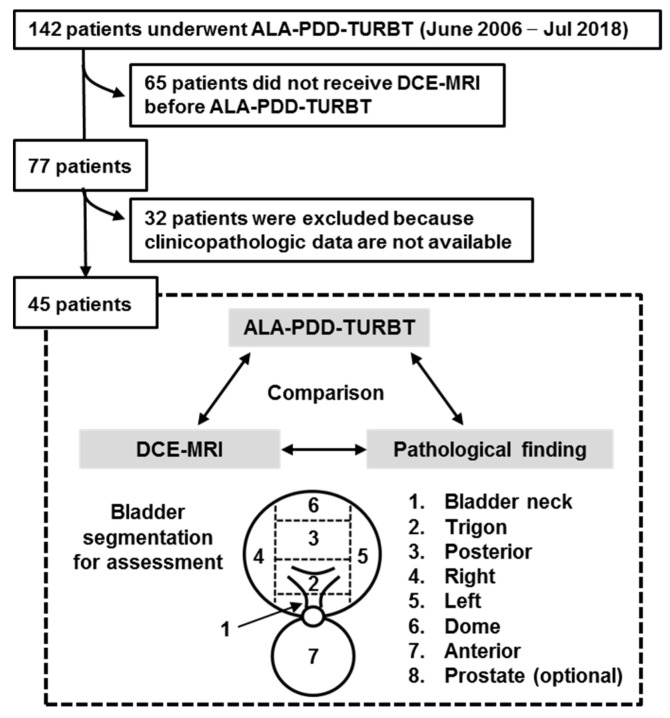
Flow chart for creation of the patient’s cohort dataset. Out of 142 patients, 45 (31.6%) met the enrollment criteria of this study. The cold-cup biopsies of the flat mucosa of all the eight segmented regions were performed and subjected to the pathological examination. A Black arrow indicate bladder neck. The test results from three different modalities were compared by the pathological finding (double arrows). ALA, 5-aminolevulinic acid; PDD, photodynamic diagnosis; TURBT, transurethral resection of the bladder tumor; DCE, dynamic contrast enhanced; MRI, magnetic resonance imaging.

Table 1 depicts the clinicopathologic variables of the 45 patients undergoing PDD-TURBT for suspected NMIBC. Out of 45 patients, 19 (42.2%) harbored pathological CIS. Nine were diagnosed concomitant CIS with a papillary tumor, while the remaining were diagnosed with pure CIS without any papillary lesion. A total of 344 biopsy specimens of flat mucosa of all the eight segmented regions were obtained and subjected to the pathological examination (Figure 2).

### 3.2. Hypervascularity and Hyperproliferation of the Bladder CIS Lesions

**Figure 3:**
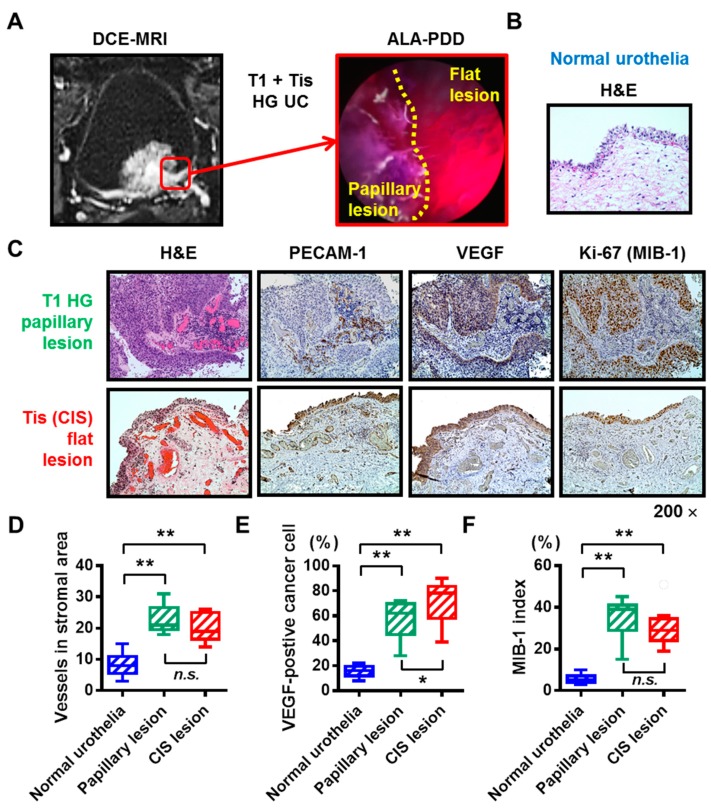
Hypervascularity and hyperproliferation of the bladder carcinoma in situ (CIS). (**A**) DCE-MRI imaging and ALA-PDD picture during TURBT of a representative case (the 73-year-old man). The red square indicates the border between the papillary tumor lesion (T1 high-grade urothelial carcinoma) and the flat tumor lesion (CIS). (**B**) H&E staining of the normal urothelia of the representative case. (**C**) All the images were captured at 200× magnification. (**D**–**F**) The matching specimens of the normal urothelial, papillary tumor lesion, and CIS from the nine patients with papillary tumor and concomitant CIS were compared with IHC analysis for PECAM-1, VEGF, and Ki-67. The number of stromal vessels, percentage of VEGF-positive cancer cells, and the percentage of proliferating cancer cells were quantified and compared. In the box-and-whisker plot or bar charts, significance (* *p* < 0.05, ** *p* < 0.01) was assessed by the paired Wilcoxon signed-rank test. CIS, carcinoma in situ; DCE, dynamic contrast enhanced; MRI, magnetic resonance imaging; ALA, 5-aminolevulinic acid; PDD, photodynamic diagnosis; TURBT, transurethral resection of the bladder tumor; *n.s.*, not significant.

To evaluate the level of hypervascularity of the bladder CIS, we carried out IHC analysis using the surgical specimens obtained from the nine patients with concomitant CIS with a papillary tumor. DCE-MRI image of the whole bladder and ALA-PDD image of the border between the papillary lesion (T1-high grade tumor) and the flat lesion (CIS) of a representative case are shown in Figure 3A. Hematoxylin and eosin (H&E) staining revealed that normal mucosa had well-organized urothelial cells and few blood vessels in the stromal area (Figure 3B). However, both papillary tumor lesion and CIS showed abundant blood vessels in the stromal area (Figure 3C). The matching specimens of the normal urothelial, papillary tumor lesion, and CIS were compared using IHC analysis for PECAM-1, VEGF, and Ki-67 (Figure 3C–F). The paired Wilcoxon signed-rank test demonstrated that CIS was characterized by both, high neovascularity and proliferation to the same level with the corresponding papillary lesion. The hypervascularity feature of the CIS lesion underlies the strong submucosal contrast-enhancement in the DCE-MRI.

### 3.3. Preoperative Urinary Cytology and Pathological CIS

A positive result of the urinary cytology suggests the presence of CIS anywhere in the urinary tract. Out of 19 patients harboring pathological CIS, 14 (73.6%) were positive and 5 (26.4%) were negative for preoperative urinary cytology. Out of 26 patients without pathological CIS, five (19.2%) were positive and 21 (80.8%) were negative for preoperative urinary cytology. In patients with Ta/T1 ≤ lesion, there is no way to tell whether the positive result was attributed from the CIS lesion. When the analysis was limited to the nine patients with pure CIS (no Ta/T1 ≤ lesion), five (55.6%) were positive and four (44.4%) were negative for preoperative urinary cytology. The detection rate was not high enough even in patients with the CIS lesion.

### 3.4. Detection of the CIS Lesions by Multiple Imaging Modalities

We set out two different methods for detecting CIS based on the combination of FL source and DCE-MRI, namely, combinations 1 and 2. Combination 1 was positive when either the FL source or DCE-MRI was test-positive. On the other hand, combination 2 was positive when both, the FL source and DCE-MRI were test-positive. The detection accuracy for the bladder CIS in WL source, FL source, DCE-MRI, and combination of FL source and DCE-MRI is listed according to the bladder segmentation (Table 2). The overall detection accuracy for bladder CIS was calculated from the 344 specimens and is listed in Table 3. The sensitivity, specificity, PPV, and NPV of each test for detecting the CIS varied over a wide range depending on the bladder location. As expected, regardless of the location, sensitivity of the FL source (average 64.3%, range, 20.0–85.7%) was higher than the WL source (average 53.6%, range 12.5–90.9%), whereas specificity of the WL source (average 88.5%, range, 82.4–95.8%) was significantly higher (*p* = 0.002) than the FL source (average 83.7%, range, 79.0–87.5%). The overall sensitivity and specificity for detecting CIS using DCE-MRI were 48.2% and 81.9%, respectively, which are the worst among the three modalities. The sensitivity for CIS of the anterior wall was 100%, whereas the sensitivity of the bladder neck and dome was significantly low (0% and 20.0%, respectively). When the FL source was combined with DCE-MRI, the overall sensitivity of the combination 1 was 75.0%, which was the best among all the detection modalities (Table 3). However, the specificity was decreased to 74.0%. As to combination 2, the sensitivity (37.5%) was much worse compared to another single modality (*p* < 0.001 when compared to FL source). However, the specificity was increased to 91.7% (*p* < 0.001 when compared to FL source).

## 4. Discussion

CIS cannot be diagnosed with imaging methods alone and requires a pathological examination. Given the poor sensitivity of the conventional WL source for detecting the bladder CIS, there is a need to develop a new method with high diagnostic accuracy. Cytological examination using a voided urine or bladder-washing specimens show high sensitivity in Grade 3 and other high-grade tumors (84%) [28], especially in CIS (28–100%) [29]. A positive result of the urinary cytology suggests the presence of CIS anywhere in the urinary tract. In our cohort, when the analysis was limited to the nine patients with pure CIS (no Ta/T1 lesion), five (55.6%) were positive and four (44.4%) were negative for preoperative urinary cytology. Random multiple bladder biopsies are performed to diagnose the suspected bladder CIS. Although the bladder CIS is characterized by reddish, coarse, and edematous urinary mucosa under the WL source, some of the lesions are small and/or have a normal appearance, thus they can be overlooked. PDD-guided TURBT or biopsy based on fluorescence excitation has been widely used to overcome this clinical limitation [3,12]. However, the significant drawbacks of PDD include false-positive results [13]. The present study addresses the potential application of DCE-MRI combined with ALA-PDD for the detection of bladder CIS. Based on our findings, the submucosal hypervascularity feature of the CIS lesion underlies the strong submucosal contrast-enhancement in the DCE-MRI (Figure 3B,C).

Here, we have reported the potential benefit of combination of DCE-MRI and ALA-PDD for CIS detection (Table 2 and Table 3). We tested two combination methods: combination 1 (either) or combination 2 (both). The overall sensitivity/specificity/PPV/NPV of the combination 1 was 75.0%/74.0%/35.9%/93.8%, respectively. The sensitivity was statistically improved as compared to ALA-PDD alone, whereas the specificity was statistically decreased and there was no change in PPV and NPV. On the other hand, the overall sensitivity/specificity/PPV/NPV of the combination 2 was 37.5%/91.7%/46.7%/88.3%, respectively. The specificity was statistically improved as compared to ALA-PDD alone, whereas the sensitivity was significantly decreased and NPV showed a marginal decrease. There is an urgent need to establish a standard protocol for diagnosis in DCE-MRI and ALA-PDD.

The multiparametric MRI-ultrasound fusion biopsy is currently an accepted modality in the pathological detection of prostate cancer [30]. In contrast to prostate cancer, little effort has been made for developing the ‘MRI-PDD fusion TURBT’ for the diagnosis and treatment of bladder cancer. In this paper, the pre-surgical segmentation model was adopted as a pilot feasibility study of ‘MRI-PDD fusion TURBT’ (Figure 1). The bladder segmentation in the DCE-MRI is required to create an accurate real-time three-dimensional mapping of the bladder. There are multiple organs in its surroundings such as the prostate, small bowel intestine, sigmoidal colon, and rectum. The deformability of the bladder and the surrounding organs make the effective segmentation difficult. Detailed consideration of both inflation and internal forces of the bladder allows to reconstruct the deformable bladder and delineate the boundaries between the bladder and its surrounding organs. However, this process is time-consuming if done manually, and usually, affected by intra- and inter-expert variabilities. Therefore, the automation of the bladder segmentation step would save time and provide the reproducibility and objectivity. Garnier et al. proposed a fast, deformable model to segment the bladder [31]. The precise algorithm developed by the evaluation of 33 MRI volumes from five different devices showed good performance, thus providing a smooth and accurate surface of the bladder.

**Figure 4:**
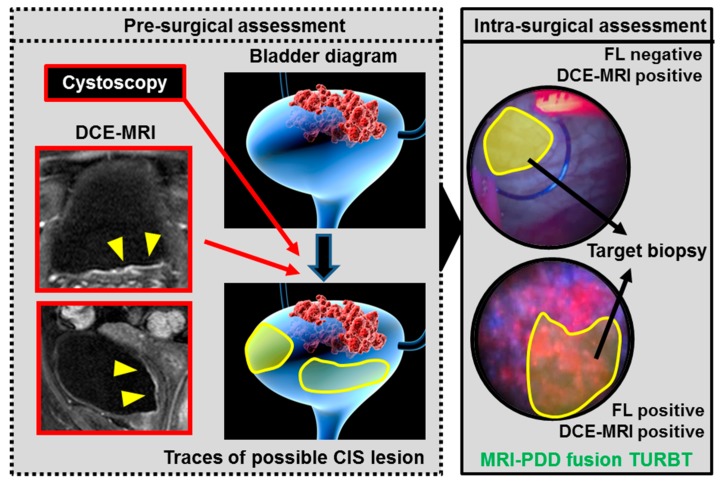
The concept of future ‘MRI-PDD fusion TURBT’. MRI-PDD fusion TURBT consists of two steps: pre-surgical assessment and intra-surgical assessment. The pre-surgical assessment requires a cystoscopic examination and DCE-MRI. During these imaging tests, the same volume of saline (for example 200 mL) is stored in the bladder through the catheter. The three-dimensional bladder diagram is reconstructed with the imaging results (red arrows). Yellow shade indicates bladder lesions suspected to be CIS based on the DCE-MRI. Surgeons need to have an informative image regarding the bladder shape and malignant lesions before the endoscopic surgery. During the surgery, the FL source provides additional information for the papillary and flat bladder lesions, especially CIS (intra-surgical assessment). The MRI-PDD fusion imaging is shown in the surgical monitor during TURBT, enabling the target biopsy for detecting CIS (black arrows).

Figure 4 shows the concept of future ‘MRI-PDD fusion TURBT’, which consists of two steps: pre-surgical assessment and intra-surgical assessment. The vital step of the pre-surgical assessment is to fuse the images of cystoscopic examination and DCE-MRI. Bladder capacity should be the same during these imaging tests. Surgeons will be able to reconstruct the three-dimensional bladder diagram before the endoscopic surgery. During the surgery, the FL source provides additional information for papillary and flat bladder lesions, especially CIS. The application of MRI-PDD fusion imaging is shown in the surgical monitor during TURBT, enabling the target biopsy. The random bladder biopsy for detecting CIS will be replaced with the target biopsy. However, specific software for MRI-PDD fusion is currently not available.

The present study has several limitations. The first is small sample size, in that only 45 patients were analyzed in this study. The second is its retrospective nature with potential selection bias—for example, some patients were excluded because of missing DCE-MRI data. Further, the radiographic review of the bladder MRI imaging was performed by a single radiologist. Further review by multiple radiologists would be able to evaluate the concordance in test results between the reviewers.

## 5. Conclusions

We explored the potential application of the DCE-MRI combined with PDD for the detection of bladder CIS. Our future goal is to establish the ‘MRI-PDD fusion TURBT’, which could be an effective therapeutic and diagnostic approach in the clinical management of high-risk NMIBC. However, further development of specific software for MRI-PDD fusion is necessary. A prospective study is required to prove a true clinical benefit and apply this novel modality to the clinical practice.

## Figures and Tables

**Table 1:** Clinicopathologic variables of 45 patients undergoing ALA-PDD-TURBT for suspected NMIBC.

Variable	*n* = 45
**Gender, *n* (%)**	
Male	41 (91%)
Female	4 (8.9%)
**Age at TURBT (years-old)**	
Mean ± SD	72 ± 8.5
Median (range)	73 (41–88)
**Pathological findings, *n* (%)**	
No malignant lesion	6 (13%)
Ta, Low grade	11 (24%)
Ta, High grade	2 (4.4%)
T1, High grade	15 (33%)
Concomitant CIS with Ta or T1 tumor	9 (20%)
Pure CIS (no papillary lesion)	9 (20%)
≥T2 (MIBC)	2 (4.4%) ^#^

ALA, 5-aminolevulinic acid; PDD, photodynamic diagnosis; TURBT, tranurethral resection of the bladder tumor; NMIBC, non-muscle invasive bladder cancer; SD, standard deviation; CIS, carcinoma in sit; ^#^ One patient had CIS.

**Table 2:** The detection accuracy for bladder CIS for the eight bladder segmentations in 45 patients undergoing PDD-TURBT for suspected NMIBC.

Patohological Finding of Biopsy Specimen	Total *n* = 45	WL Source		FL Source (ALA-PDD)		DCE-MRI		FL and DCE-MRI Combination 1 ^†^		FL and DCE-MRI Combination 2 ^‡^	
		Positive	Negative	Positive	Negative	Positive	Negative	Positive	Negative	Positive	Negative
**1. Bladder neck**											
CIS-positive	4	2	2	3	1	0	4	3	1	0	4
CIS-negative	41	3	38	4	37	9	32	11	30	2	39
Sensitivity		50.0% (8.8–91.1)		75.0% (30.0–98.7)		0.0% (0.0–49.0)		75.0% (30.1–98.7)		0.0% (0.0–49.0)	
Specificity		92.7% (80.6–97.5)		90.2% (77.5–96.1)		78.1% (63.3–88.0)		73.2% (58.1–84.3)		95.2% (83.9–99.1)	
PPV		40.0% (71.1–76.9)		42.9% (15.8–75.0)		0.0% (0.0–29.9)		21.4% (7.6–47.6)		0.0% (0.0–82.2)	
NPV		95.0% (83.5–99.1)		97.4% (86.5–99.9)		88.9% (74.7–95.6)		96.8% (83.8–99.8)		90.7% (78.4–96.3)	
**2. Trigon (including ureteral orifice)**											
CIS-positive	7	6	1	6	1	7	0	7	0	6	1
CIS-negative	38	10	28	13	25	14	24	20	18	7	31
Sensitivity		85.7% (48.7–99.3)		85.7% (48.7–99.3)		100% (64.6–100)		100% (64.6–100)		85.7% (48.7–99.3)	
Specificity		73.7% (58.0–85.0)		65.8% (49.9–78.8)		63.2% (47.3–76.6)		47.3% (32.5–62.7)		81.6% (66.6–90.8)	
PPV		37.5% (18.5–61.4)		31.6% (15.4–54.0)		33.3% (17.2–54.6)		25.9% (13.2–44.7)		46.2% (23.2–70.9)	
NPV		96.6% (82.8–99.8)		96.2% (81.1–99.8)		100% (86.2–100)		100% (82.4–100)		96.9% (84.3–99.8)	
**3. Posterior**											
CIS-positive	11	10	1	9	2	6	5	10	1	5	6
CIS-negative	34	6	28	12	22	7	27	14	20	5	29
Sensitivity		90.9% (62.3–99.5)		81.8% (52.3–96.8)		54.6% (28.0–78.7)		90.9% (62.3–99.5)		45.5% (21.3–72.0)	
Specificity		82.4% (66.5–91.7)		64.7% (47.9–78.5)		79.4% (63.2–89.7)		58.8% (42.2–73.6)		85.3% (69.9–93.6)	
PPV		62.6% (38.6–81.5)		42.9% (24.5–63.5)		46.2% (23.2–70.7)		41.7% (24.5–61.2)		50.0% (23.7–76.3)	
NPV		96.6% (82.8–99.8)		91.7% (74.2–98.5)		84.4% (68.3–93.1)		95.2% (77.3–99.8)		82.9% (67.3–91.9)	
**4. Right**											
CIS-positive	9	6	3	6	3	5	4	7	2	4	5
CIS-negative	36	3	33	3	33	5	31	5	31	3	33
Sensitivity		66.7% (35.4–87.9)		66.7% (35.4–87.9)		55.6% (26.7–81.1)		77.8% (45.3–96.1)		44.4% (18.9–73.3)	
Specificity		91.7% (78.2–97.1)		91.7% (78.2–97.1)		86.1% (71.3–93.9)		86.1% (71.3–93.9)		91.7% (78.2–97.1)	
PPV		66.7% (35.4–87.9)		66.7% (35.4–87.9)		50.0% (23.7–76.3)		58.3% (32.0–80.7)		57.1% (25.1–84.2)	
NPV		91.7% (78.2–97.1)		91.7% (78.2–97.1)		88.6% (74.1–95.5)		93.9% (80.4–98.9)		86.8% (72.7–94.3)	
**5. Left**											
CIS-positive	8	1	7	4	4	2	6	4	4	2	6
CIS-negative	37	4	33	5	32	5	32	8	29	2	35
Sensitivity		12.5% (0.6–47.1)		50.0% (21.5–78.5)		25.0% (44.4–59.1)		50.0% (21.5–78.5)		25.0% (4.4–59.1)	
Specificity		89.2% (75.3–95.7)		86.5% (72.0–94.1)		86.5% (72.0–94.1)		78.4% (62.8–88.6)		94.6% (82.3–99.0)	
PPV		20.0% (1.0–62.5)		44.4% (18.9–73.3)		28.6% (50.8–64.1)		33.3% (13.8–60.9)		50.0% (8.9–91.1)	
NPV		82.5% (68.1–91.3)		88.9% (74.7–95.6)		84.2% (69.6–92.6)		87.9% (72.7–95.2)		85.4% (71.6–93.1)	
**6. Dome**											
CIS-positive	5	3	2	4	1	1	4	4	1	1	4
CIS-negative	40	3	37	3	37	4	36	5	35	2	38
Sensitivity		60.0% (23.1–92.9)		80.0% (37.6–99.0)		20.0% (1.0–62.5)		80.0% (37.6–99.0)		20.0% (10.3–62.5)	
Specificity		92.5% (80.1–97.4)		92.5% (80.1–97.4)		90.0% (77.0–96.0)		82.5% (73.9–94.5)		95.0% (83.5–99.1)	
PPV		50.0% (18.8–81.2)		57.1% (25.1–84.2)		20.0% (0.10–62.5)		44.4% (18.9–73.3)		33.3% (1.7–88.2)	
NPV		94.9% (83.1–99.1)		97.4% (86.5–99.9)		90.0% (77.0–96.0)		97.2% (85.8–99.9)		90.5% (77.9–96.2)	
**7. Anterior wall**											
CIS-positive	7	1	6	3	4	4	3	4	3	3	4
CIS-negative	38	3	35	4	34	2	36	5	33	1	37
Sensitivity		14.3% (0.7–51.4)		42.9% (15.8–75.0)		57.1% (25.1–84.2)		57.1% (25.1–84.2)		42.9% (15.8–75.0)	
Specificity		92.1% (79.2–97.3)		89.5% (75.9–95.8)		94.7% (82.7–99.1)		86.8% (72.7–94.3)		97.4% (86.5–99.9)	
PPV		25.0% (1.3–69.9)		42.9% (15.8–75.0)		66.8% (30.0–94.1)		44.4% (18.9–73.3)		75.0% (30.1–98.7)	
NPV		85.4% (71.6–93.1)		89.5% (75.9–95.8)		92.3% (79.7–97.4)		91.7% (78.2–97.1)		90.2% (77.5–96.1)	
**8. Prostatic urethra (opitional, *n* = 29)**											
CIS-positive	5	1	4	1	4	2	3	3	2	0	5
CIS-negative	24	1	23	3	21	6	18	7	17	2	22
Sensitivity		20.0% (1.0–62.5)		20.0% (1.0–62.5)		40.0% (7.1–76.9)		60.0% (23.1–92.9)		0.0% (0.0–43.5)	
Specificity		95.8% (79.8–99.8)		87.5% (69.0–95.7		75.0% (5.5–88.0)		70.8% (50.8–85.1)		91.7% (74.2–98.5)	
PPV		50.0% (2.6–97.4)		25.0% (1.3–69.9)		25.0% (4.4–59.1)		30.0% (10.8–60.3)		0.0% (0.0–82.2)	
NPV		85.2% (67.5–94.1)		84.0% (65.4–93.6)		85.7% (65.4–95.0)		89.5% (68.6–98.1)		81.5% (63.3–91.8)	

The ranges in parenthesis are 95% confidence intervals of the diagnostic value. CIS, carcinoma in situ; PDD, photodynamic diagnosis; TURBT, transurethral resection of the bladder tumor; ALA, 5-aminolevulinici acid; WL, white-light; FL, fluorescence; DCE-MRI, dynamic contrast-enhanced magnetic resonance imaging; ND, not determined; PPV, positive predictive value; NPV, negative predictive value; ^†^ the combination 1 is positive when either the FL source or DCE-MRI was test-positive; ^‡^ the combination 2 is positive only when both FL source and DCE-MRI are test-positive.

**Table 3:** The overall detection accuracy for bladder CIS in 45 patients undergoing PDD-TURBT for suspected NMIBC.

Patohological Finding of Biopsy Specimen	Total *n* = 344	WL Source		FL Source (ALA-PDD)		DCE-MRI		FL and DCE-MRI Combination 1 ^†^		FL and DCE-MRI Combination 2 ^‡^		*p* Value		
		Positive	Negative	Positive	Negative	Positive	Negative	Positive	Negative	Positive	Negative	WL Source *vs* FL Source	FL Source *vs* Combination 1	FL Source *vs* Combination 2
**CIS-positive**	56	30	26	36	20	27	29	42	14	21	35			
**CIS-negative**	288	33	255	47	241	52	236	75	213	24	264			
**Sensitivity**		53.6% (40.7–66.0)		64.3% (51.2–75.5)		48.2% (35.7–61.0)		75.0% (62.3–84.5)		37.5% (26.0–50.6)		0.15 ^#^	0.041 ^#^	< 0.001 ^#^
**Specificity**		88.5% (84.3–91.7)		83.7% (79.0–87.5)		81.9% (77.1–86.0)		74.0% (68.6–78.7)		91.7% (87.9–94.3)		0.002 ^#^	< 0.001 ^#^	< 0.001 ^#^
**PPV**		47.6% (35.8–59.7)		43.4% (33.2–54.1)		34.2% (24.5–45.2)		35.9% (27.8–44.9)		46.7% (32.9–60.9)		0.70 ^##^	0.74 ^##^	0.59 ^##^
**NPV**		90.8% (86.8–93.6)		92.3% (88.5–95.0)		89.1% (84.7–92.3)		93.8% (89.9–96.3)		88.3% (84.2–91.5)		0.48 ^##^	0.41 ^##^	0.08 ^##^

The ranges in parenthesis are 95% confidence intervals of the diagnostic value. CIS, carcinoma in situ; PDD, photodynamic diagnosis; TURBT, transurethral resection of the bladder tumor; ALA, 5-aminolevulinici acid; WL, white-light; FL, fluorescence; DCE-MRI, dynamic contrast-enhanced magnetic resonance imaging; ND, not determined; PPV, positive 8predictive value; NPV, negative predictive value; ^†^ the combination 1 is positive when either the FL source or DCE-MRI was test-positive; ^‡^ the combination 2 is positive only when both FL source and DCE-MRI are test-positive, ^#^ McNemar’s test; ^##^ Chi-square test.
